# Vedolizumab treatment for immune checkpoint inhibitor-induced enterocolitis

**DOI:** 10.1007/s00262-017-1962-6

**Published:** 2017-02-15

**Authors:** Viktoria Bergqvist, Erik Hertervig, Peter Gedeon, Marija Kopljar, Håkan Griph, Sara Kinhult, Ana Carneiro, Jan Marsal

**Affiliations:** 1 Department of Gastroenterology, Skane University Hospital, 22185 Lund, Sweden; 2 Immunology Section, Department of Experimental Medical Science, Lund University, Lund, Sweden; 3 Section of Medicine, Department of Clinical Sciences, Lund University, Lund, Sweden; 4 Department of Oncology, Skane University Hospital, Lund, Sweden; 5 Division of Oncology and Pathology, Department of Clinical Sciences, Lund University, Lund, Sweden; 6 Department of Respiratory Medicine, Skane University Hospital, Lund, Sweden

**Keywords:** Ipilimumab, Nivolumab, Melanoma, Lung cancer, Immune-checkpoint inhibitor induced enterocolitis, Vedolizumab treatment against irAEs

## Abstract

Immune checkpoint inhibitors (ICPI), such as ipilimumab [anti-cytotoxic T-lymphocyte antigen-4 (CTLA-4) antibody] and nivolumab or pembrolizumab [anti-programmed cell death protein-1 (PD-1) antibodies], improve survival in several cancer types. Since inhibition of CTLA-4 or PD-1 leads to non-selective activation of the immune system, immune-related adverse events (irAEs) are frequent. Enterocolitis is a common irAE, currently managed with corticosteroids and, if necessary, anti-tumor necrosis factor-α therapy. Such a regimen carries a risk of serious side-effects including infections, and may potentially imply impaired antitumor effects. Vedolizumab is an anti-integrin α4β7 antibody with gut-specific immunosuppressive effects, approved for Crohn’s disease and ulcerative colitis. We report a case series of seven patients with metastatic melanoma or lung cancer, treated with vedolizumab off-label for ipilimumab- or nivolumab-induced enterocolitis, from June 2014 through October 2016. Clinical, laboratory, endoscopic, and histologic data were analyzed. Patients initially received corticosteroids but were steroid-dependent and/or partially refractory. One patient was administered infliximab but was refractory. The median time from onset of enterocolitis to start of vedolizumab therapy was 79 days. Following vedolizumab therapy, all patients but one experienced steroid-free enterocolitis remission, with normalized fecal calprotectin. This was achieved after a median of 56 days from vedolizumab start, without any vedolizumab-related side-effects noted. The patient in whom vedolizumab was not successful, due to active ulcerative colitis, received vedolizumab prophylactically. This is the first case series to suggest that vedolizumab is an effective and well-tolerated therapeutic for steroid-dependent or partially refractory ICPI-induced enterocolitis. A larger prospective study to evaluate vedolizumab in this indication is warranted.

## Introduction

Immune checkpoint inhibitors (ICPIs) have greatly improved possibilities for treatment in oncology. The physiological role of immune checkpoints, such as cytotoxic T-lymphocyte antigen-4 (CTLA-4) and programmed cell death protein-1 (PD-1) which are expressed primarily on T cells, is to regulate the degree of T cell activation, to prevent exaggerated immune responses or autoimmunity [1]. CTLA-4 is thought to control initial T cell activation and proliferation in lymph nodes early in an immune response. In contrast, PD-1 regulates T cell activity later in an immune response, at the effector site, i.e. the tumor [1]. Antibodies blocking CTLA-4 (ipilimumab) or PD-1 (nivolumab and pembrolizumab) thus augment the immunological response to tumors through related but distinct mechanisms. Furthermore, it is thought that antibodies to CTLA-4 induce depletion of regulatory T cells which express CTLA-4 [1, 2]. These new drugs have shown significant survival benefits in advanced melanoma, non-small cell lung cancer, renal cancer, and squamous head and neck cancer, and their efficacy is being investigated in several other cancers [1, 3].

ICPIs expand and/or activate not only tumor-specific T cells but also numerous other T cell clones. This may break down peripheral self-tolerance and cause immune-related adverse events (irAEs). ICPIs have been shown to be associated with a number of irAEs, such as enterocolitis, hepatitis, rash, vitiligo, and hypophysitis [4, 5]. Most irAEs are manageable but they can occasionally be very severe and even fatal as in cases with enterocolitis that leads to intestinal perforation [6–8]. Severe or life-threatening irAEs (grade 3–4) are more common with CTLA-4 blockers (reported rates of 20–27%) than with PD-1 blockers (13–16%), whereas with combination treatment the rate rises to around 50% [3, 8]. The most common grade 3–4 irAEs with all of the various ICPI regimes are gastrointestinal (i.e. diarrhea, enterocolitis, and hepatitis) [4, 5].

Currently, irAEs are managed in accordance with algorithms that are broadly used but not evidence based [9]. Mild (grade 1) diarrhea/enterocolitis can be managed with symptomatic therapy, but in moderate and severe cases (grade 2–3), corticosteroids are used [4, 5]. If corticosteroids are not sufficient, i.e. lack of response to high-dose corticosteroids (2 mg/kg methylprednisolone) within days, more potent immunosuppressive agents such as infliximab [anti-tumor necrosis factor (TNF) α antibody], mycophenylate mofetil, or calcineurin inhibitors are recommended [5].

The level of inflammation in the gut can be measured in several ways. The traditional way is to quantify symptoms, primarily the number of bowel movements per day. This parameter is used to grade ICPI-induced diarrhea. A drawback with using symptoms for quantifying inflammation is that they are indirect and subjective in character. Colonoscopy is the gold standard but is cumbersome to perform repeatedly. An even more stringent measure is the histology of the gut mucosa. Plasma CRP and albumin are used but may be affected by other systemic inflammatory processes. A relatively new measure of gut inflammation, that has high sensitivity and specificity, is the fecal (F) concentration of calprotectin. Calprotectin is a calcium-binding protein derived primarily from neutrophils and activated macrophages [10]. Levels of F-calprotectin have been shown to correlate closely with gastrointestinal inflammation [11].

Blood lymphocyte counts have shown a positive correlation, and the blood neutrophil/lymphocyte ratio (NLR) a negative correlation, with overall survival in several solid tumor diseases, both before and after the onset of ICPI therapy, and both with and without corticosteroid therapy taken into account [12, 13]. Corticosteroids are known to induce apoptosis in lymphocytes but not neutrophils [14] and to induce demargination of neutrophils from the endovascular lining. Similarly, infliximab functions by inducing apoptosis of activated T-lymphocytes through transmembrane TNFα, in addition to inhibiting soluble TNFα [15].

Vedolizumab is a humanized monoclonal IgG1 antibody against α4β7 integrin, approved for treatment of Crohn’s disease and ulcerative colitis, collectively called inflammatory bowel diseases (IBD) [16, 17]. α4β7 integrin is expressed primarily on a subset of CD4^+^ T cells and mediates homing of these cells specifically to the gut [18, 19]. The counter-receptor of α4β7, mucosal addressin cellular adhesion molecule 1 (MAdCAM-1), is expressed selectively on venule endothelial cells of the gut but not in other organs [18, 20]. Blocking of α4β7 integrin thus results in a gut-specific immunosuppression, while the immunity in extraintestinal tissues remains intact [20]. In addition, vedolizumab is specific for the dimer α4β7 and does not bind to other integrins that comprise the α4 or β7 subunit, such as α4β1 which is important for the homing of lymphocytes to a number of organs [19]. The gut-specific effect of vedolizumab is further illustrated by the fact that there have not been any reports of progressive multifocal leukoencephalopathy (PML) with vedolizumab, in contrast to another integrin-inhibitor, natalizumab, which targets α4β1 in addition to α4β7, and thus inhibits lymphocyte function systemically [21]. It is well recognized that vedolizumab has a gradual onset of therapeutic effect [22]. This may be explained by the mode-of-action which is inhibition of recruitment of new lymphocytes to the gut mucosa, with less effect on the cells already present in the inflamed gut tissue [20].

Here, we report on seven patients with ICPI-induced enterocolitis, which were either corticosteroid-dependent and/or partially refractory, managed successfully with vedolizumab infusions.

## Methods

Seven patients who had received immunotherapy with either ipilimumab or nivolumab against advanced melanoma (*n* = 6) or non-small cell lung cancer (*n* = 1) between January 2014 and July 2015 at Skane University Hospital, received subsequent treatment with vedolizumab due to IPCI-induced enterocolitis (here defined as inflammation primarily of the colon and distal ileum with or without confirmed inflammation in more proximal segments). The first of these patients was started on vedolizumab therapy in June 2014 and the last in June 2016. The therapy decisions were clinically based and taken by a multidisciplinary team comprising gastroenterologists and oncologists. The patients were, retrospectively, identified and enrolled for review and analysis of medical records. The study was approved by the regional ethics committee in Lund, Sweden (reference No. 2016/624). No patient consent was required as this was a retrospective study. The study was performed in accordance with the ethical standards of the 1964 Helsinki declaration and its later amendments.

All laboratory blood and fecal tests were taken as part of clinical routine. F-calprotectin was measured using a commercially available enzyme-linked immunosorbent assay (ELISA) (Calpro AS). The lower limit of the ELISA was 30 mg/kg; values below this cut-off were estimated as 15 mg/kg. F-calprotectin levels <50 mg/kg are normal according to the manufacturer, but studies in IBD have suggested 100 mg/kg to be a more adequate cut-off level [23].

The degree of diarrhea was graded 1 to 5 in accordance with the Common Terminology Criteria for Adverse Events version 4.0 which grades the increase in number of stools per day above the baseline value [24]: Grade 1, increase of <4 stools; grade 2, 4–6 stools; grade 3, ≥7 stools; grade 4, diarrhea with life-threatening consequences; grade 5, death. Films and pictures from the ileocolonoscopic examinations recorded for clinical purposes, were categorized blindly by an experienced endoscopist into normal, mild, moderate, or severe inflammation, based on global assessment of the most inflamed segment with regards to several parameters including erythema, vascular pattern, granularity, bleeding, and ulceration. In addition, the ileocolonoscopic examinations were scored using the Ulcerative Colitis Endoscopic Index of Severity (UCEIS) [25] and the Simple Endoscopic Score for Crohn’s Disease (SES-CD) [26], respectively. These scores were used since a specific endoscopic index for immune-therapy associated enterocolitis does not exist, as yet. The score range for a single intestinal segment is 0–8 for UCEIS and 0–12 for SES-CD, and 0–20 for the combination of these. Thresholds defining remission, mild, moderate, and severe disease have yet to be established. However, for UCEIS, it has been suggested that a score of 0–1 represents remission and 7–8 represents severe disease in the context of ulcerative colitis, whereas the cut-off separating mild from moderate disease remains unclear [27]. Colonic mucosal histopathology was categorized into normal, mild, moderate, or severe inflammation based on global assessment of several parameters including acute and chronic inflammatory infiltration of lamina propria and the epithelium, and architectural disorder.

Performance status was assessed in accordance with the Eastern Cooperative Oncology Group (ECOG) scale ranging from 0 to 5 [28]. Statistical analysis was performed using Prism 6 for Mac OS X version 6.0h (GraphPad Software, Inc.).

## Results

### Patient characteristics

Five patients diagnosed with metastatic melanoma (stage IV) and one with melanoma stage IIIc were treated with ipilimumab. One patient with non-small cell lung cancer stage IV was treated with nivolumab. Patient characteristics are summarized in Table 1. At initiation of ICPI therapy, patient age ranged from 40 to 71 years with a median of 55 years. The male to female ratio was 4:3. Performance status according to the ECOG scale was 0 for three patients and 1 for four patients at initiation of therapy.

**Table 1:** Patient characteristics and cancer therapy

Case no.	Age (years)	Gender	Other diseases	Current malignancy		Performance status acc. ECOG	Immune therapy			Previous therapy	Radiotherapy
				Diagnosis	Stage		Drug	Dosing (mg/kg)	Number of infusions		
1	68	M	–	Malignant melanoma	IV	0	Ipilimumab	3	2	–	After ICPI therapy
2	71	M	Hypertension^a,^ prostate cancer^b,^ deep vein thrombosis^b^, cerebrovascular accident^b^	Malignant melanoma	IV	1	Ipilimumab	3	4	Nivolumab	Prior to ICPI therapy*
3	48	F	Ulcerative colitis^a^	Malignant melanoma	IV	1	Ipilimumab	3	4	Temozolomide, pembrolizumab	Prior to ICPI therapy*
4	64	F	–	Malignant melanoma	IV	0	Ipilimumab	3	4	Temozolomide, dacarbazine	–
5	55	F	Cervical cancer^b^	Malignant melanoma	IV	1	Ipilimumab	3	2	–	Concomitant with ICPI therapy*
6	40	M	–	Malignant melanoma	IIIc	0	Ipilimumab	10	2	–	–
7	55	M	Crohn’s disease^b^, pulmonary embolism^b^, atrial fibrillation^b^, chronic obstructive pulmonary disease III^a^, sarcoidosis^b^	Non-small cell lung cancer	IV	1	Nivolumab	3	18	Carboplatin, gemcitabine, taxotere	–

*ICPI* immune checkpoint inhibitor, *M* Male, *F* female, *ECOG* Eastern Cooperative Oncology Group scale [28]

*No radiation to the abdominal organs

^a^Comorbidities

^b^Previous diseases

Two patients had a history of inflammatory bowel disease. Patient No. 3 had a history of ulcerative colitis that increased in activity after treatment with pembrolizumab. Before this patient was switched to ipilimumab because of tumor progression, she was started on prophylactic vedolizumab treatment. Patient No. 7 had undergone a right hemicolectomy due to Crohn’s disease in adolescence, which led to sustained inflammatory remission, and showed no signs of inflammatory bowel disease when nivolumab therapy was started. This patient had previously also been diagnosed with atrial fibrillation, pulmonary embolism, sarcoidosis and chronic obstructive pulmonary disease. Patients No. 2 and No. 5 had a history of prostate and cervical cancer, respectively.

### Cancer therapy

Ipilimumab or nivolumab were dosed at 3 mg/kg of body weight with an interval of 3 weeks for ipilimumab and 2 weeks for nivolumab, in all patients except for patient No. 6 who was given 10 mg/kg body weight of ipilimumab every 3 weeks (Table 1). Between infusions 1 and 2, patient No. 5 received radiation therapy against axillary lymph nodes with 25 Gy in 5 fractions. Four patients had previously received chemotherapy and/or another type of immunotherapy (Table 1). The number of infusions given before onset of enterocolitis symptoms ranged from 2 to 4 for patients receiving ipilimumab, whereas the patient on nivolumab therapy received 18 doses prior to symptom development (Table 1). ICPI therapy was discontinued in all patients upon development of grade 3 enterocolitis with grade 2–3 diarrhea, and the total number of infusions hence equals the number of infusions given before symptom onset.

### Diagnosis, management, and evaluation of ICPI-induced enterocolitis

The median time that elapsed from the first dose of ipilimumab to onset of enterocolitis symptoms was 65 days (range 38–88 days) (Table 2). The median time from the last dose to development of symptoms was 19 days (range 9–27 days) (Table 2). Patient No. 7 who received 18 nivolumab infusions did not develop enterocolitis until 292 days after therapy was commenced. Two patients presented with grade 2 diarrhea, and five patients with grade 3 diarrhea (Table 2). Patient No. 5 developed additional immune-related adverse events (irAEs) in the form of rash and iritis, but in the other patients diarrhea/enterocolitis were the only irAEs requiring treatment. Bacterial cause for diarrhea was ruled out by means of stool cultures and *Clostridium difficile* toxin tests. At diagnosis, all patients were examined by computed tomography scanning. Large and/or small bowel wall thickening was present in five cases, and in two cases the scans was considered inconclusive.

**Table 2:** Immune checkpoint inhibitor-induced enterocolitis characteristics and vedolizumab therapy

Case no.	Days to onset of diarrhea		Maximal diarrhea grade acc. CTCAE 4	Other immune-related adverse events	Response to corticosteroids	Previous infliximab therapy	Inflammation		Days from enterocolitis onset to vedolizumab start	Prednisolone dose at vedolizumab start (mg/day)	Vedolizumab therapy			
	from the first ICPI infusion	from the last ICPI infusion					Acc. endoscopy	Acc. histology			Dosing (mg)	Number of infusions	Side effects	Days from start to steroid-free remission
1	38	16	3	–	Dependent	No	Mild	Mild	86	40	300	2	No	52
2	80	17	3	–	Partially refractory	No	Moderate	Moderate	82	100	300	2	No	53
3	88	25	3	–	Partially refractory and dependent	No	Moderate	Moderate	*	*	300	4	No	*
4	85	22	3	–	Dependent	No	Moderate	Severe	61	20	300	2	No	57
5	48	27	2	Rash, iritis	Partially refractory and dependent	No	Moderate	Mild	84	40	300	2	No	65
6	51	9	2	–	Dependent	No	Mild	Mild	76	70	300	4	No	56
7	292	11	3	–	Dependent	Yes	Moderate	Severe	57	160	300	3	No	92

*ICPI* immune checkpoint inhibitor, *CTCAE 4* Common Terminology Criteria for Adverse Events version 4.0 [24]

*Received vedolizumab prophylactically prior to ipilimumab

The patients were initially treated with corticosteroids in accordance with international recommendations for treatment of IPCI-induced enterocolitis [4, 5], including intravenous administration of methylprednisolone dosed up to 2 mg/kg body weight. The enterocolitis that these patients displayed was either partially steroid-refractory (i.e. partial but not complete response) and/or steroid-dependent (i.e. at adequate tapering of high-dose corticosteroids, patients exhibited increased signs of enterocolitis).

**Fig. 1:**
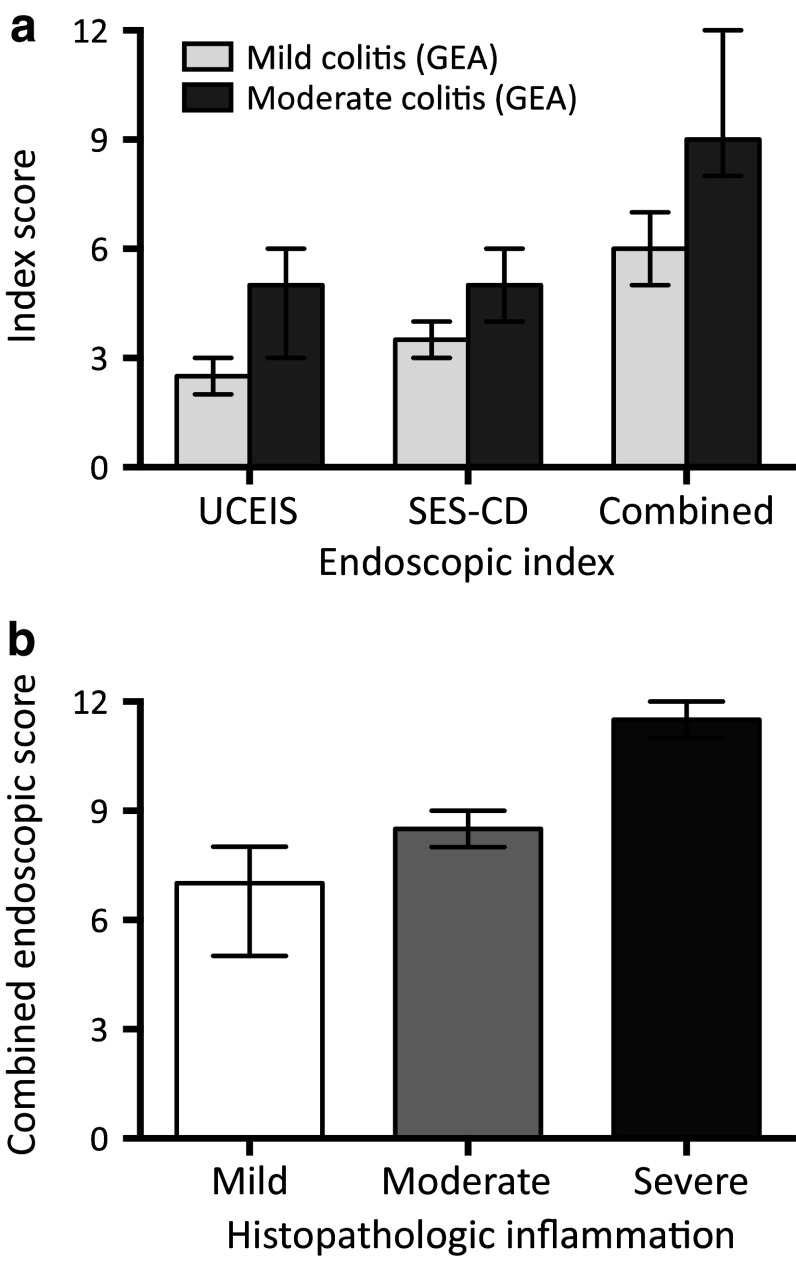
Evaluation of immune checkpoint inhibitor-induced enterocolitis using endoscopic scores developed for Crohn’s disease and ulcerative colitis. **a** Colonoscopy findings were categorized based on global endoscopic assessment (GEA) of the most inflamed segment (normal, mild, moderate, or severe inflammation), and compared to endoscopic index scores generated using the Ulcerative Colitis Endoscopic Index of Severity (UCEIS; 0–8 points) [25], Simple Endoscopic Score for Crohn’s Disease (SES-CD; 0–12 points) [26], and a combined endoscopic score (sum of individual UCEIS and SES-CD scores; 0–20 points). **b** Colonic mucosal histopathology was categorized into normal, mild, moderate, or severe inflammation, and compared to the combined endoscopic score. *Columns* and *error bars* represent median values ± range

Prior to starting vedolizumab therapy, all patients underwent ileocolonoscopy. Endoscopic inflammatory signs were scored in accordance with the UCEIS and SES-CD indexes and an integrated global inflammation categorization was performed. Two patients displayed mild endoscopic inflammation and five displayed moderate inflammation, according to the global endoscopic assessment (Table 2). The mean UCEIS, SES-CD, and combined scores for patients with mild or moderate endoscopic findings are shown in Fig. 1a. Histopathologic evaluation of colonic mucosal biopsies showed mild inflammation in three cases, moderate inflammation in two cases, and severe inflammation in two cases (Table 2). The combined endoscopic scores corresponded well to the histopathologic findings as shown in Fig. 1b.

### Vedolizumab treatment for ICPI-induced enterocolitis

When treated for their ICPI-induced diarrhea/enterocolitis, patients No. 1, 4, 6, and 7 responded to high-dose corticosteroids but were considered steroid-dependent as they relapsed when therapy was tapered in accordance with international recommendations (i.e. tapering scheduled over 4–8 weeks). Patients No. 2, 3, and 5 responded only partially to high-dose corticosteroids and were thus considered partially steroid-refractory (Table 2). Inadequate inflammatory control on corticosteroid therapy despite optimized dosing and repeated retapering attempts, prompted the use of vedolizumab. The median time from onset of enterocolitis to start of vedolizumab was 79 days (range 57–86) (Table 2). Vedolizumab was administered with infusions of 300 mg at time-points 0, 2, and 6 weeks or until clinical and laboratory regression was observed (Table 2). This corresponds to the induction treatment regimen recommended for Crohn’s disease and ulcerative colitis. Patient No. 7 initially received infliximab therapy but since this was unsuccessful, vedolizumab was administered. At the start of vedolizumab treatment, patient No. 1, 2, 4, 5, and 6 displayed grade 1, and patient No. 7 grade 3 diarrhea. In addition, patient No. 6 and 7 had abdominal pain. The number of vedolizumab infusions given was 2 to 4; at the time of the first infusion, the daily dose of prednisolone ranged from 20 to 160 mg with a mean of 78 mg (Table 2). Prednisolone could then be successfully tapered in all of the patients except in patient No. 3. The median time from start of vedolizumab treatment to steroid-free remission from enterocolitis symptoms was 56 days (range 52–92 days). No side effects related to vedolizumab treatment were observed (Table 2).

Patient No. 3 had mildly active ulcerative colitis before initiation of ipilimumab therapy and was given vedolizumab for prophylactic purpose 1 week prior to initiation of ipilimumab infusions to avoid potential aggravation of the underlying colitis disease, as opposed to induction therapy against ICPI-induced enterocolitis. Unfortunately, diarrheal symptoms increased markedly after the fourth ipilimumab dose and thus the desired protective effect from vedolizumab was not achieved.

**Fig. 2:**
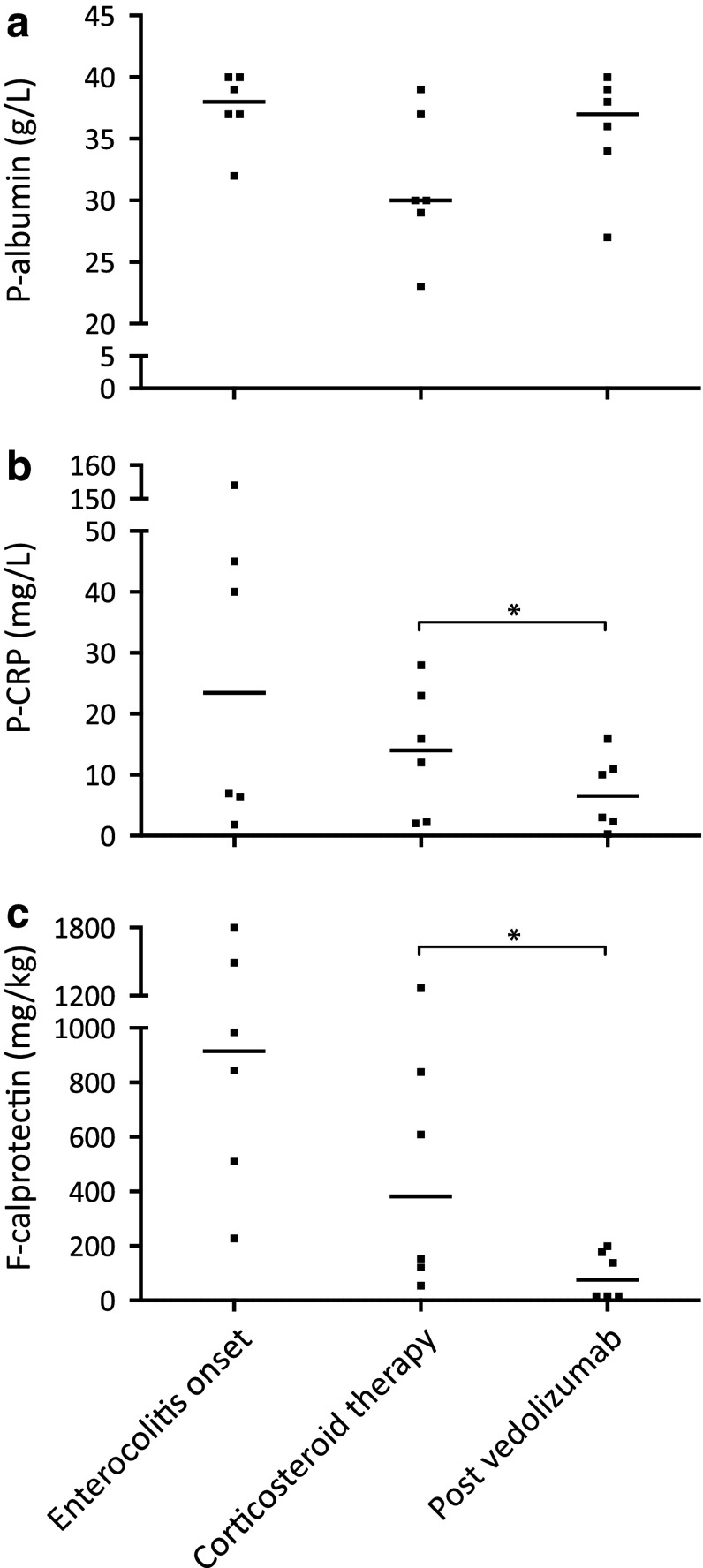
Laboratory inflammatory biomarkers in patients with immune checkpoint inhibitor-induced enterocolitis, in relation to corticosteroid and vedolizumab treatment. **a** Plasma albumin concentrations at enterocolitis onset, during corticosteroid therapy just before starting vedolizumab, and after vedolizumab therapy. **b** Plasma C-reactive protein concentrations at corresponding time-points. **c** Fecal calprotectin concentrations at corresponding time-points. *Enterocolitis onset*: at onset of ICPI-induced enterocolitis, *Corticosteroid therapy*: just prior to initiation of vedolizumab treatment, *Post vedolizumab*: after vedolizumab treatment and after corticosteroids had been tapered. *P* plasma, *F* fecal, *CRP* C-reactive protein, *ICPI* immune checkpoint inhibitor. Individual values and medians; *P < 0.05 (Wilcoxon test); *n* = 6 (patient No. 3, who received vedolizumab prophylactically, was excluded from this analysis)

**Fig. 3:**
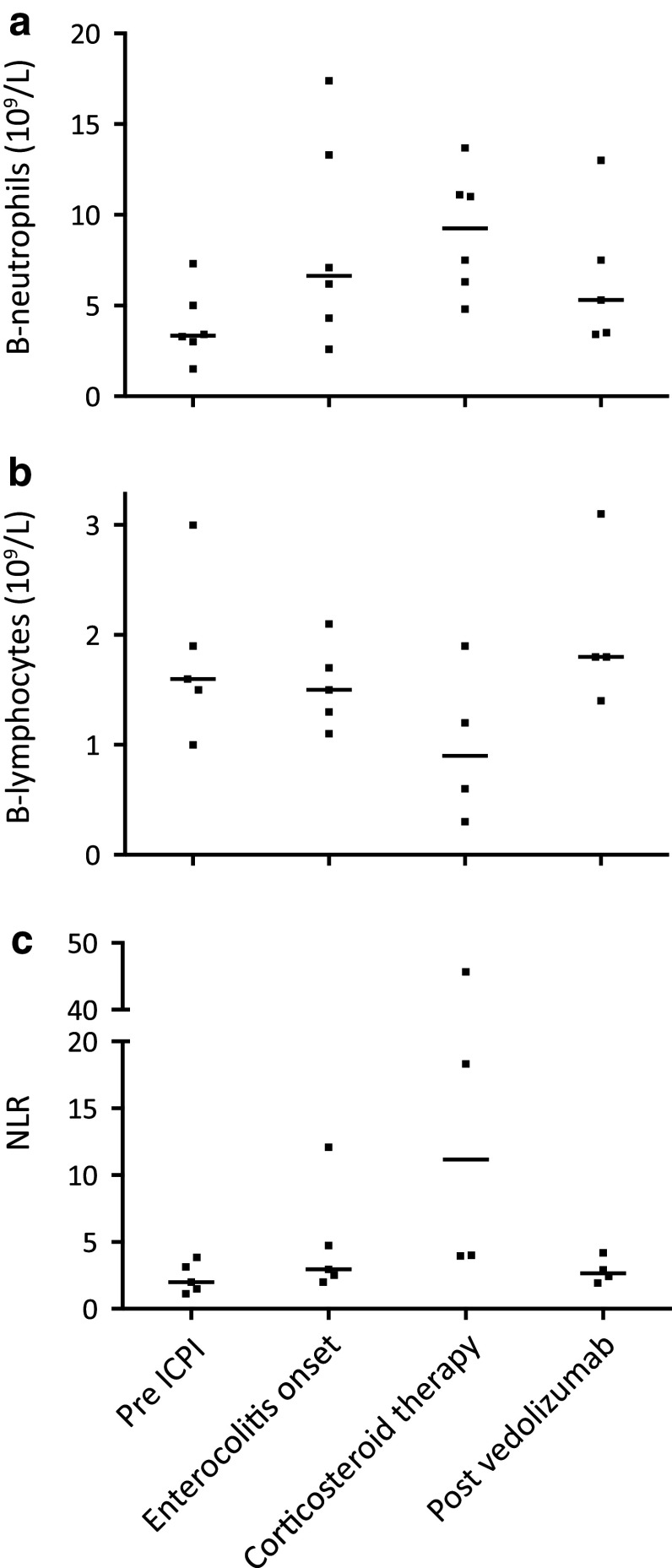
Blood lymphocyte and neutrophil counts in patients with immune checkpoint inhibitor-induced enterocolitis, in relation to ICPI, corticosteroid, and vedolizumab treatment. **a** Blood neutrophil counts before onset of enterocolitis, at enterocolitis onset, during corticosteroid therapy just before starting vedolizumab, and after vedolizumab therapy. **b** Blood lymphocyte counts at corresponding time-points. **c** The blood neutrophil/lymphocyte ratio at corresponding time-points. Individual values and medians. Blood cell counts were not available in all patients. *Pre ICPI*: just prior to initiation of ICPI therapy, *Enterocolitis onset*: at onset of ICPI-induced enterocolitis, *Corticosteroid therapy*: just prior to initiation of vedolizumab treatment, *Post vedolizumab*: after vedolizumab treatment and after corticosteroids had been tapered. *B* blood, *ICPI* immune checkpoint inhibitor, *NLR* neutrophil/lymphocyte ratio

Routine laboratory tests, including plasma (P) C-reactive protein (CRP), P-albumin, blood (B) neutrophils, B-lymphocytes, and F-calprotectin, were taken in all patients before, during, and after the periods when ICPI therapy and vedolizumab, respectively, were administered (Figs. 2, 3). Patient No. 3 was excluded from the analyses presented in Figs. 2 and 3 since vedolizumab in this case was given prophylactically (see above).

Median P-CRP and F-calprotectin were increased at the onset of diarrhea to 23.5 mg/L (range 1.8–154.0) and 915 mg/kg (range 228–1799), respectively, and decreased as a result of treatment with corticosteroids to 14.0 mg/L (range 2.0–28.0) and 382 mg/kg (range 54–1268), respectively. Of note, after vedolizumab treatment, the levels of P-CRP and F-calprotectin decreased even further to 6.5 mg/L (range 0.3–16.0) and 76 mg/kg (range 15–199) (Fig. 2b, c). In contrast, P-albumin levels decreased after treatment with corticosteroids (median 30 g/L; range 23–39) and increased after administration of vedolizumab (median 37 g/L; range 27–40) (Fig. 2a).

Examination of blood lymphocyte levels and the neutrophil/lymphocyte ratio (NLR) showed decreased levels of lymphocytes and an increase in the NLR after patients had been treated with corticosteroids (Fig. 3b, c). After vedolizumab therapy, these parameters were normalized (Fig. 3b, c).

## Discussion

We report on our experience of vedolizumab as an effective and safe therapy for treating ICPI-induced enterocolitis in selected patients, based on a case series of seven patients. These results corroborate a recent report on a single patient who had developed colitis secondary to ipilimumab therapy and was successfully treated with vedolizumab [29]. The patients selected for vedolizumab therapy in our retrospective study were either steroid-dependent and/or partially steroid refractory. Management in accordance with established algorithms with corticosteroids was applied but tapering was not feasible in these patients [5]. Additional immunosuppressive therapy was required to control the enterocolitis, and these patients thus represent a group of difficult-to-treat cases. This is underscored by the fact that the patients were on futile corticosteroid therapy for a median of 79 days before vedolizumab therapy was started. However, since all patients were on corticosteroids when vedolizumab therapy was initiated, the gut inflammation and symptoms were generally mild to moderate at this point. We considered the most adequate read-out parameter with regards to vedolizumab efficacy to be steroid-free enterocolitis remission. Our findings suggest that administration of two to four infusions of vedolizumab is sufficient to achieve steroid-free remission. In our patients, this endpoint was reached within a median of 56 days from the start of vedolizumab treatment. This may be compared to recommendations advocating the use of infliximab together with a steroid-taper over 45–60 days or more in cases of steroid refractory ICPI-induced enterocolitis [4]. Considering the exhaustive attempts to control the inflammation with corticosteroids including high doses and repeated unsuccessful tapering, it is very unlikely that the inflammation would have resolved without additional immunosuppressive therapy. Vedolizumab was chosen for this purpose as infliximab was considered excessive in relation to irAE severity; moreover, there is an unclear relationship between anti-TNFα therapy and melanoma [30–33].

The diagnosis of ICPI-induced enterocolitis was confirmed endoscopically and histopathologically in all patients. It is well known that the correlation between the degree of gastrointestinal symptoms and the level of gut inflammation is often poor. Therefore, gastroenterologists aim at using structured and objective measures of inflammation for evaluation and monitoring of inflammatory gut disorders. Since ICPI-induced enterocolitis may exhibit features typical for both Crohn’s disease and/or ulcerative colitis, we combined two validated scores for Crohn’s disease (SES-CD) and ulcerative colitis (UCEIS), respectively, when evaluating enterocolitis in these patients. Interestingly, the combined score corresponded better to the histopathological degree of inflammation (Fig. 1b), than the global endoscopic assessment did (data not shown), suggesting that development of a specific endoscopic index for ICPI-induced enterocolitis would be useful for a more accurate grading of the inflammation. This, in turn, would be helpful in choosing and evaluating appropriate anti-inflammatory therapies.

In addition to enabling corticosteroid tapering, vedolizumab treatment led to a notable reduction in the levels of F-calprotectin and P-CRP, reflecting the attenuated inflammation in the gut. This was supported by a normalizing trend in P-albumin. The changes observed in blood cell counts were expected, given known effects of corticosteroids causing lymphopenia and neutrophilia [14].

There were no vedolizumab-related adverse events among our patients, which is in agreement with the documented favorable safety profile of vedolizumab. Results from six clinical trials for IBD comprising 2830 patients and 4811 person-years of vedolizumab exposure have not shown any increased risks of infections, serious infections including PML, malignancies, or infusion-related reactions over placebo [16, 17, 21]. Among the various immunosuppressants, systemic corticosteroids show the strongest association with serious infections, but the risk increases steeply when systemically immunsuppressive agents are combined [34–37]. It seems that serious infections in the context of infliximab therapy for irAEs are relatively rare, but nevertheless potentially fatal. There are case reports on the subject [38–40], and a retrospective study of 740 ICPI-treated patients with melanoma found the rate of serious infections to be 7.3% and identified corticosteroids and/or infliximab as the main risk factors [37].

Treatment with infliximab is recommended in cases of corticosteroid refractory ICPI-induced enterocolitis [4, 41, 42]. In a recent study on 254 melanoma patients with irAEs, 29 (11.4%) patients received infliximab therapy and high-dose corticosteroids [43]. Among these 29 patients, 21 patients responded to infliximab but 8 (27.6%) did not respond and received prolonged courses of corticosteroids. There is very little data available on the effects and safety of infliximab treatment in this context and on the potential effects of corticosteroids in combination with infliximab on the anti-tumor efficacy of ICPI therapy [42]. In a retrospective study on ipilimumab-treated melanoma patients who had developed diarrhea, there was no significant difference in the median overall survival time between those treated with infliximab (*n* = 7) and those treated with corticosteroids only (*n* = 29) [44]. However, an important aspect omitted in this type of analysis is that the median overall survival time of the infliximab-treated patient group could have been even longer had they not received infliximab. This question could be addressed in studies utilizing vedolizumab. There is an ongoing debate whether irAEs and/or the severity of irAEs is associated with better cancer-specific outcomes, and whether treatment with immunosuppressive agents potentially counteracts the anti-tumor effect of ICPI therapy. There are several studies supporting these concepts [3, 44–49] but also those refuting them [5, 43, 44, 48, 50–52].

We believe that vedolizumab is an appropriate choice of therapy for many but not all patients with ICPI-induced enterocolitis. For instance, patients with extraintestinal irAEs may require systemic immunosuppression rather than vedolizumab which has a gut-specific effect. In our material, only one out of seven patients (14%) had additional irAEs, and in a larger published cohort, this proportion was 37% [53]. As illustrated by patient No. 7, vedolizumab may also be used as second-line therapy in infliximab-refractory cases, which is true in IBD as well [22]. In addition, vedolizumab may be considered when contraindications to infliximab prevail or when infliximab is not tolerated. In one of the patients, vedolizumab was administered prophylactically to prevent a flare of preexisting and mildly active UC. Unfortunately, this was not successful; this could speak against vedolizumab being efficacious in this type of scenario. But given that this was a single case, the concept of prophylactic vedolizumab therapy in patients with known IBD should be explored in larger studies. Interestingly, there are reports on patients with IBD who received ipilimumab without increased disease activity [54]. Other situations where prophylactic treatment with vedolizumab could be suggested are (a) combination treatment with ipilimumab and a PD-1 inhibitor where the risk of severe enterocolitis is increased [3], and (b) adjuvant ICPI therapy in high-risk resected melanoma where the acceptance for irAEs may be decreased given an apparent lack of malignant disease [9]. Taken together, patients that we consider suitable for vedolizumab treatment are those with steroid-dependent and/or partially refractory mild to moderate enterocolitis, and without extraintestinal irAEs that require additional immunosuppression. In contrast, patients with severe gut inflammation that demands urgent measures should be considered for other therapies such as infliximab or, as last resort, colectomy.

In conclusion, this is the first case series to suggest that vedolizumab is an effective and well-tolerated therapy for steroid refractory and/or dependent enterocolitis caused by treatment with ipilimumab or nivolumab. In addition to these, and pembrolizumab, there are several new ICPI agents being subjected to clinical evaluation in numerous types of malignancies, and subsequently the prevalence of ICPI-induced enterocolitis may be expected to increase considerably in the near future. Thus, there is a growing need for developing evidence-based and increasingly optimized irAE management algorithms, taking the long-term cancer-treatment strategy and the overall survival into account.

## Abbreviations

B: Blood
CRP: C-reactive protein
CTLA-4: Cytotoxic T-lymphocyte antigen-4
ECOG: Eastern cooperative oncology group
ELISA: Enzyme-linked immunosorbent assay
F: Fecal
IBD: Inflammatory bowel diseases
ICPI: Immune checkpoint inhibitor
irAE: Immune-related adverse event
MAdCAM-1: Mucosal addressin cellular adhesion molecule-1
NLR: Neutrophil/lymphocyte ratio
P: Plasma
PD-1: Programmed cell death protein-1
PML: Progressive multifocal leukoencephalopathy
SES-CD: Simple endoscopic score for Crohn’s disease
TNF: Tumor necrosis factor
UCEIS: Ulcerative colitis endoscopic index of severity

## References

[1] Buchbinder EI, Desai A (2016). CTLA-4 and PD-1 pathways: similarities, differences, and implications of their inhibition. Am J Clin Oncol.

[2] Romano E, Kusio-Kobialka M, Foukas PG, Baumgaertner P, Meyer C, Ballabeni P, Michielin O, Weide B, Romero P, Speiser DE (2015). Ipilimumab-dependent cell-mediated cytotoxicity of regulatory T cells ex vivo by nonclassical monocytes in melanoma patients. Proc Natl Acad Sci USA.

[3] Larkin J, Chiarion-Sileni V, Gonzalez R, Grob JJ, Cowey CL, Lao CD, Schadendorf D, Dummer R, Smylie M, Rutkowski P, Ferrucci PF, Hill A, Wagstaff J, Carlino MS, Haanen JB, Maio M, Marquez-Rodas I, McArthur GA, Ascierto PA, Long GV, Callahan MK, Postow MA, Grossmann K, Sznol M, Dreno B, Bastholt L, Yang A, Rollin LM, Horak C, Hodi FS, Wolchok JD (2015). Combined nivolumab and ipilimumab or monotherapy in untreated melanoma. N Engl J Med.

[4] Weber JS, Kahler KC, Hauschild A (2012). Management of immune-related adverse events and kinetics of response with ipilimumab. J Clin Oncol.

[5] Spain L, Diem S, Larkin J (2016). Management of toxicities of immune checkpoint inhibitors. Cancer Treat Rev.

[6] Kwon ED, Drake CG, Scher HI, Fizazi K, Bossi A, van den Eertwegh AJ, Krainer M, Houede N, Santos R, Mahammedi H, Ng S, Maio M, Franke FA, Sundar S, Agarwal N, Bergman AM, Ciuleanu TE, Korbenfeld E, Sengelov L, Hansen S, Logothetis C, Beer TM, McHenry MB, Gagnier P, Liu D, Gerritsen WR, Investigators CA (2014). Ipilimumab versus placebo after radiotherapy in patients with metastatic castration-resistant prostate cancer that had progressed after docetaxel chemotherapy (CA184-043): a multicentre, randomised, double-blind, phase 3 trial. Lancet Oncol.

[7] Eggermont AM, Chiarion-Sileni V, Grob JJ, Dummer R, Wolchok JD, Schmidt H, Hamid O, Robert C, Ascierto PA, Richards JM, Lebbe C, Ferraresi V, Smylie M, Weber JS, Maio M, Konto C, Hoos A, de Pril V, Gurunath RK, de Schaetzen G, Suciu S, Testori A (2015). Adjuvant ipilimumab versus placebo after complete resection of high-risk stage III melanoma (EORTC 18071): a randomised, double-blind, phase 3 trial. Lancet Oncol.

[8] Robert C, Schachter J, Long GV, Arance A, Grob JJ, Mortier L, Daud A, Carlino MS, McNeil C, Lotem M, Larkin J, Lorigan P, Neyns B, Blank CU, Hamid O, Mateus C, Shapira-Frommer R, Kosh M, Zhou H, Ibrahim N, Ebbinghaus S, Ribas A, investigators K- (2015). Pembrolizumab versus Ipilimumab in advanced melanoma. N Engl J Med.

[9] Spain L, Larkin J (2016). Weighing up the pros and cons of immune checkpoint inhibitors in the treatment of melanoma. Immunotherapy.

[10] Poullis A, Foster R, Mendall MA, Fagerhol MK (2003). Emerging role of calprotectin in gastroenterology. J Gastroenterol Hepatol.

[11] Langhorst J, Elsenbruch S, Koelzer J, Rueffer A, Michalsen A, Dobos GJ (2008). Noninvasive markers in the assessment of intestinal inflammation in inflammatory bowel diseases: performance of fecal lactoferrin, calprotectin, and PMN-elastase, CRP, and clinical indices. Am J Gastroenterol.

[12] Templeton AJ, McNamara MG, Seruga B, Vera-Badillo FE, Aneja P, Ocana A, Leibowitz-Amit R, Sonpavde G, Knox JJ, Tran B, Tannock IF, Amir E (2014). Prognostic role of neutrophil-to-lymphocyte ratio in solid tumors: a systematic review and meta-analysis. J Natl Cancer Inst.

[13] Callahan MK, Wolchok JD, Allison JP (2010). Anti-CTLA-4 antibody therapy: immune monitoring during clinical development of a novel immunotherapy. Semin Oncol.

[14] Gruver-Yates AL, Cidlowski JA (2013). Tissue-specific actions of glucocorticoids on apoptosis: a double-edged sword. Cells.

[15] Levin AD, Wildenberg ME, van den Brink GR (2016). Mechanism of action of anti-TNF therapy in inflammatory bowel disease. J Crohns Colitis.

[16] Sandborn WJ, Feagan BG, Rutgeerts P, Hanauer S, Colombel JF, Sands BE, Lukas M, Fedorak RN, Lee S, Bressler B, Fox I, Rosario M, Sankoh S, Xu J, Stephens K, Milch C, Parikh A, Group GS (2013). Vedolizumab as induction and maintenance therapy for Crohn’s disease. N Engl J Med.

[17] Feagan BG, Rutgeerts P, Sands BE, Hanauer S, Colombel JF, Sandborn WJ, Van Assche G, Axler J, Kim HJ, Danese S, Fox I, Milch C, Sankoh S, Wyant T, Xu J, Parikh A, Group GS (2013). Vedolizumab as induction and maintenance therapy for ulcerative colitis. N Engl J Med.

[18] Berlin C, Berg EL, Briskin MJ, Andrew DP, Kilshaw PJ, Holzmann B, Weissman IL, Hamann A, Butcher EC (1993). Alpha 4 beta 7 integrin mediates lymphocyte binding to the mucosal vascular addressin MAdCAM-1. Cell.

[19] Soler D, Chapman T, Yang LL, Wyant T, Egan R, Fedyk ER (2009). The binding specificity and selective antagonism of vedolizumab, an anti-alpha4beta7 integrin therapeutic antibody in development for inflammatory bowel diseases. J Pharmacol Exp Ther.

[20] Wyant T, Fedyk E, Abhyankar B (2016). An Overview of the mechanism of action of the monoclonal antibody vedolizumab. J Crohns Colitis.

[21] Colombel JF, Sands BE, Rutgeerts P, Sandborn W, Danese S, D’Haens G, Panaccione R, Loftus EV, Sankoh S, Fox I, Parikh A, Milch C, Abhyankar B, Feagan BG (2016). The safety of vedolizumab for ulcerative colitis and Crohn’s disease. Gut.

[22] Bryant RV, Sandborn WJ, Travis SP (2015). Introducing vedolizumab to clinical practice: who, when, and how?. J Crohns Colitis.

[23] von Roon AC, Karamountzos L, Purkayastha S, Reese GE, Darzi AW, Teare JP, Paraskeva P, Tekkis PP (2007). Diagnostic precision of fecal calprotectin for inflammatory bowel disease and colorectal malignancy. Am J Gastroenterol.

[24] Common Terminology Criteria for Adverse Events (CTCAE), Version 4.0. (U.S. Department of Health and Human Services; National Institutes of Health; National Cancer Institute; 2010). http://evs.nci.nih.gov/ftp1/CTCAE/CTCAE_4.03_2010-06-14_QuickReference_8.5x11.pdf

[25] Travis SP, Schnell D, Krzeski P, Abreu MT, Altman DG, Colombel JF, Feagan BG, Hanauer SB, Lichtenstein GR, Marteau PR, Reinisch W, Sands BE, Yacyshyn BR, Schnell P, Bernhardt CA, Mary JY, Sandborn WJ (2013). Reliability and initial validation of the ulcerative colitis endoscopic index of severity. Gastroenterology.

[26] Daperno M, D’Haens G, Van Assche G, Baert F, Bulois P, Maunoury V, Sostegni R, Rocca R, Pera A, Gevers A, Mary JY, Colombel JF, Rutgeerts P (2004). Development and validation of a new, simplified endoscopic activity score for Crohn’s disease: the SES-CD. Gastrointest Endosc.

[27] Travis SP, Schnell D, Feagan BG, Abreu MT, Altman DG, Hanauer SB, Krzeski P, Lichtenstein GR, Marteau PR, Mary JY, Reinisch W, Sands BE, Schnell P, Yacyshyn BR, Colombel JF, Bernhardt CA, Sandborn WJ (2015). The impact of clinical information on the assessment of endoscopic activity: characteristics of the ulcerative colitis endoscopic index of severity [UCEIS]. J Crohns Colitis.

[28] Oken MM, Creech RH, Tormey DC, Horton J, Davis TE, McFadden ET, Carbone PP (1982). Toxicity and response criteria of the eastern cooperative oncology group. Am J Clin Oncol.

[29] Hsieh AH, Ferman M, Brown MP, Andrews JM (2016). Vedolizumab: a novel treatment for ipilimumab-induced colitis. BMJ Case Rep 2016.

[30] Long MD, Martin CF, Pipkin CA, Herfarth HH, Sandler RS, Kappelman MD (2012). Risk of melanoma and nonmelanoma skin cancer among patients with inflammatory bowel disease. Gastroenterology.

[31] Mariette X, Matucci-Cerinic M, Pavelka K, Taylor P, van Vollenhoven R, Heatley R, Walsh C, Lawson R, Reynolds A, Emery P (2011). Malignancies associated with tumour necrosis factor inhibitors in registries and prospective observational studies: a systematic review and meta-analysis. Ann Rheum Dis.

[32] Raaschou P, Simard JF, Holmqvist M, Askling J, Group AS (2013). Rheumatoid arthritis, anti-tumour necrosis factor therapy, and risk of malignant melanoma: nationwide population based prospective cohort study from Sweden. BMJ.

[33] Wolfe F, Michaud K (2007). Biologic treatment of rheumatoid arthritis and the risk of malignancy: analyses from a large US observational study. Arthritis Rheum.

[34] Bongartz T, Sutton AJ, Sweeting MJ, Buchan I, Matteson EL, Montori V (2006). Anti-TNF antibody therapy in rheumatoid arthritis and the risk of serious infections and malignancies: systematic review and meta-analysis of rare harmful effects in randomized controlled trials. JAMA.

[35] Marehbian J, Arrighi HM, Hass S, Tian H, Sandborn WJ (2009). Adverse events associated with common therapy regimens for moderate-to-severe Crohn’s disease. Am J Gastroenterol.

[36] Toruner M, Loftus EV, Harmsen WS, Zinsmeister AR, Orenstein R, Sandborn WJ, Colombel JF, Egan LJ (2008). Risk factors for opportunistic infections in patients with inflammatory bowel disease. Gastroenterology.

[37] Del Castillo M, Romero FA, Arguello E, Kyi C, Postow MA, Redelman-Sidi G (2016). The spectrum of serious infections among patients receiving immune checkpoint blockade for the treatment of melanoma. Clin Infect Dis.

[38] Lord JD, Hackman RC, Moklebust A, Thompson JA, Higano CS, Chielens D, Steinbach G, McDonald GB (2010). Refractory colitis following anti-CTLA4 antibody therapy: analysis of mucosal FOXP3 + T cells. Dig Dis Sci.

[39] Kyi C, Hellmann MD, Wolchok JD, Chapman PB, Postow MA (2014). Opportunistic infections in patients treated with immunotherapy for cancer. J Immunother Cancer.

[40] Arriola E, Wheater M, Krishnan R, Smart J, Foria V, Ottensmeier C (2015). Immunosuppression for ipilimumab-related toxicity can cause pneumocystis pneumonia but spare antitumor immune control. Oncoimmunology.

[41] Beck KE, Blansfield JA, Tran KQ, Feldman AL, Hughes MS, Royal RE, Kammula US, Topalian SL, Sherry RM, Kleiner D, Quezado M, Lowy I, Yellin M, Rosenberg SA, Yang JC (2006). Enterocolitis in patients with cancer after antibody blockade of cytotoxic T-lymphocyte-associated antigen 4. J Clin Oncol.

[42] Pages C, Gornet JM, Monsel G, Allez M, Bertheau P, Bagot M, Lebbe C, Viguier M (2013). Ipilimumab-induced acute severe colitis treated by infliximab. Melanoma Res.

[43] Horvat TZ, Adel NG, Dang TO, Momtaz P, Postow MA, Callahan MK, Carvajal RD, Dickson MA, D’Angelo SP, Woo KM, Panageas KS, Wolchok JD, Chapman PB (2015). Immune-related adverse events, need for systemic immunosuppression, and effects on survival and time to treatment failure in patients with melanoma treated with ipilimumab at memorial sloan kettering cancer center. J Clin Oncol.

[44] Arriola E, Wheater M, Karydis I, Thomas G, Ottensmeier C (2015). Infliximab for IPILIMUMAB-related colitis-letter. Clin Cancer Res.

[45] Attia P, Phan GQ, Maker AV, Robinson MR, Quezado MM, Yang JC, Sherry RM, Topalian SL, Kammula US, Royal RE, Restifo NP, Haworth LR, Levy C, Mavroukakis SA, Nichol G, Yellin MJ, Rosenberg SA (2005). Autoimmunity correlates with tumor regression in patients with metastatic melanoma treated with anti-cytotoxic T-lymphocyte antigen-4. J Clin Oncol.

[46] Yang JC, Hughes M, Kammula U, Royal R, Sherry RM, Topalian SL, Suri KB, Levy C, Allen T, Mavroukakis S, Lowy I, White DE, Rosenberg SA (2007). Ipilimumab (anti-CTLA4 antibody) causes regression of metastatic renal cell cancer associated with enteritis and hypophysitis. J Immunother.

[47] Yousaf N, Davidson M, Goode E, Thomas C, Hung R, Gore M, Larkin J (2015). The cost of ipilimumab toxicity: a single-centre analysis. Melanoma Res.

[48] Downey SG, Klapper JA, Smith FO, Yang JC, Sherry RM, Royal RE, Kammula US, Hughes MS, Allen TE, Levy CL, Yellin M, Nichol G, White DE, Steinberg SM, Rosenberg SA (2007). Prognostic factors related to clinical response in patients with metastatic melanoma treated by CTL-associated antigen-4 blockade. Clin Cancer Res.

[49] Feng Y, Roy A, Masson E, Chen TT, Humphrey R, Weber JS (2013). Exposure-response relationships of the efficacy and safety of ipilimumab in patients with advanced melanoma. Clin Cancer Res.

[50] De Felice KM, Gupta A, Rakshit S, Khanna S, Kottschade LA, Finnes HD, Papadakis KA, Loftus EV, Raffals LE, Markovic SN (2015). Ipilimumab-induced colitis in patients with metastatic melanoma. Melanoma Res.

[51] Prieto PA, Yang JC, Sherry RM, Hughes MS, Kammula US, White DE, Levy CL, Rosenberg SA, Phan GQ (2012). CTLA-4 blockade with ipilimumab: long-term follow-up of 177 patients with metastatic melanoma. Clin Cancer Res.

[52] Wolchok JD, Neyns B, Linette G, Negrier S, Lutzky J, Thomas L, Waterfield W, Schadendorf D, Smylie M, Guthrie T, Grob JJ, Chesney J, Chin K, Chen K, Hoos A, O’Day SJ, Lebbe C (2010). Ipilimumab monotherapy in patients with pretreated advanced melanoma: a randomised, double-blind, multicentre, phase 2, dose-ranging study. Lancet Oncol.

[53] Verschuren EC, van den Eertwegh AJ, Wonders J, Slangen RM, van Delft F, van Bodegraven A, Neefjes-Borst A, de Boer NK (2016). Clinical, endoscopic, and histologic characteristics of ipilimumab-associated colitis. Clin Gastroenterol Hepatol.

[54] Johnson DB, Sullivan RJ, Ott PA, Carlino MS, Khushalani NI, Ye F, Guminski A, Puzanov I, Lawrence DP, Buchbinder EI, Mudigonda T, Spencer K, Bender C, Lee J, Kaufman HL, Menzies AM, Hassel JC, Mehnert JM, Sosman JA, Long GV, Clark JI (2016). Ipilimumab therapy in patients with advanced melanoma and preexisting autoimmune disorders. JAMA Oncol.

